# Esophageal Squamous Cell Carcinoma and Gastric Cardia Adenocarcinoma Shared Susceptibility Locus in PLCE1: A Meta-Analysis

**DOI:** 10.1371/journal.pone.0069214

**Published:** 2013-07-18

**Authors:** Ruiqin Mai, Yabin Cheng, Yuanshen Huang, Guohong Zhang

**Affiliations:** 1 Department of Laboratory Medicine, the First Affiliated Hospital of Shantou University Medical College, Shantou, Guangdong, China; 2 Department of Dermatology and Skin Science, University of British Columbia, Vancouver, British Columbia, Canada; 3 Department of Pathology, Shantou University Medical College, Shantou, Guangdong, China; University de Minho, Portugal

## Abstract

**Background And Objective:**

Two recent genome-wide association studies have identified a shared susceptibility variation PLCE1 rs2274223 for esophageal squamous cell carcinoma (ESCC) and gastric cardia adenocarcinomas (GCA). Subsequent case-control studies have reported this association in other populations. However, the findings were controversial and the effect remains undetermined. Our aim is to provide a precise quantification of the association between PLCE1 rs2274223 variation and the risk of ESCC and GCA.

**Methods:**

Studies were identified by a literature search in MEDLINE and EMBASE databases. Pooled odds ratios (ORs) with 95% confidence intervals (CIs) were used to assess the association in allele, dominant, recessive, homozygous, and heterozygous models.

**Results:**

Ten articles were identified, including 22156 ESCC cases and 28803 controls, 5197 GCA cases and 17613 controls. Overall, PLCE1 rs2274223 G allele (G vs. A: OR=1.26, 95% CI: 1.15-1.39 for ESCC; OR=1.51, 95% CI: 1.35–1.69 for GCA) and its carrier (GG +AG vs. AA: OR = 1.23; 95% CI =1.02-1.49 for ESCC; OR =1.62; 95% CI =1.15-2.29 for GCA) were significantly associated with the risk of ESCC and GCA. In stratified analysis by ethnicity, significant association of PLCE1 rs2274223 G allele and the risk of ESCC (OR=1.33, 95% CI 1.21–1.45) and GCA (OR =1.56, 95% CI: 1.47-1.64) was observed in Chinese population.

**Conclusions:**

Our meta-analysis results indicated that PLCE1 rs2274223 G allele significantly contributed to the risk of ESCC and GCA, especially in Chinese population.

## Introduction

Esophageal cancer is the eighth most common cancer worldwide, with approximate 482,300 new cases and 406,800 related deaths in 2008 [1], and the incidence of which varies significantly according to geographic locations and ethnicity [2]. Particularly, esophageal squamous cell carcinoma (ESCC) predominates in South Africa, South America and an area that extends from the border of the Caspian Sea and Turkey through the southern republics of the former Soviet Union and into northern China, which is often referred to as the “esophageal cancer belt” [2]. Gastric cardia adenocarcinoma (GCA) is another common type of cancer, which shares similar geographic distribution and environmental risk factors with ESCC in China [3,4]. In some ESCC high-incidence areas, ESCC and GCA are the most prevalent cancers making up around 50% of total cancer cases, and cause more than 20% of all cancer-related deaths [5]. However, in the same high-incidence area, only a subset of individuals exposed to the environmental risk factors would develop ESCC or GCA, suggesting a role of genetic variations in ESCC and GCA carcinogenesis. Previous evidences of population genetics and familial aggregation suggested that inherited susceptibility contributed significantly to the high rate of ESCC and GCA in these areas [6–8]. Furthermore, coincidence of two cancers and a family history of ESCC/GCA significantly increased the risk for both cancers, supporting a shared genetic predisposition between ESCC and GCA [9].

Recently, two independent genome-wide association studies (GWAS) in Chinese subjects simultaneously reported that a novel variant (A5780G, rs2274223) in the phospholipase C epsilon gene (PLCE1) gene was strongly associated with ESCC and GCA [10,11]. The finding was later confirmed by another independent GWAS research in Chinese population [12]. Following study showed of more cells with PLCE1 expression in ESCC and GCA tissues than in normal tissues, which further supports the idea that PLCE1 contributes to ESCC and GCA [10]. Located on 10q23, PLCE1 gene encodes a novel ras-related protein (R-Ras) effector mediating the effects of R-Ras on the actin cytoskeleton and membrane protrusion, and is involved in the regulation of cell growth, differentiation, apoptosis and angiogenesis [13,14]. PLCE1 has been reported as an oncogene in skin, intestinal and bladder carcinogenesis through inflammation signalling pathways [13]. The variant rs2274223 is a nonsynonymous SNP located in exon 26 of PLCE1, which causes an amino acid change from histidine to arginine (His1927Arg) in the calcium-dependent lipid-binding (C2) domain of PLCE1 protein.

Additional studies confirming the associated locus in other populations are essential to our understanding of the pathogenesis of ESCC and GCA. Several studies characterized and replicated the PLCE1 rs2274223 alteration in South Africa, Caucasian and Asian population for ESCC and GCA after GWAS [15–21]. But the results were generally inconsistent and inconclusive, probably due to the small size in each study. To summarize the effect of PLCE1 rs2274223 in ESCC and GCA risk, we combined risk estimate data from different populations and performed a meta-analysis.

## Materials and Methods

### Literature search and identification of eligible studies

Published articles that explored the association of PLCE1 and ESCC or GCA in peer-reviewed journals were identified by searching PubMed and Ovid MEDLINE database and ISI Web of Science, Science Citation Index Expanded, and China National Knowledge Infrastructure (CNKI) (up to Nov 1, 2012). The literature search and quantitative analyses were in accordance with following guidelines for meta-analysis of observation studies [22]. The following search terms: “oesophageal cancer”, “esophageal cancer”, “esophageal squamous cell carcinoma”, “ESCC”, “gastric adenocarcinomas”, “gastric cancer” and “PLCE1” or “phospholipase C epsilon gene” were used as well as their combinations. Only studies involving human subjects were included. There was no restriction on geographical location of studies. We also searched the manuscripts and the supplementary documents of the published GWAS in the field. Population-based case-control studies reporting associations between the PLCE1 and ESCC or GCA were included, all of which met the following criteria: (i) unrelated case-control design, (ii) genotype distribution of control population must be in Hardy-Weinberg equilibrium (HWE), and (iii) sufficient allele or genotype data for calculating odds ratios (ORs) and 95% confidence intervals (95% CI). Review articles, case-only articles, esophageal adenocarcinomas, gastric non-cardia adenocarcinoma case-control, and repeated literatures were excluded. In case of overlapping articles, only the publications with the most extensive information were included.

After database searching, we screened all titles and abstracts for their potential eligibility. Specifically, titles and abstracts were included if they indicated that it was a case-control study on PLCE1 polymorphism. The list of all titles and abstracts identified provided a pool of PLCE1 polymorphism studies. In the next stage, reviewers identified all studies that provided the most relevant data for the meta-analysis. Full copies of articles were retrieved following initial screening, then the full text of the candidate articles were examined carefully to determine whether they accorded with the inclusion criteria for the meta-analysis by Zhang GH and Mai RQ, independently.

### Data extraction

A standard data extraction form was used for data extraction from eligible publications. Data were extracted independently by two researchers from each article, including: first author’s surname, year of publication, country of origin, cancer type, and genome-wide association study or not, characteristics of cancer cases and controls, and total number of cases and controls, allele frequencies of cases, and controls information.

### Meta-analysis

The risk of ESCC and GCA associated with the PLCE1 rs2274223 variant was indicated by pooled OR with 95% CI. First, we evaluated the risk of the variant G allele, compared with the A allele (G vs. A). Heterogeneity across studies was evaluated by Cochran’s Q test and I^2^, which represents the percentage of total variation across studies that is attributable to heterogeneity rather than to chance. Heterogeneity was considered statistically significant when P < 0.05, then the pooled OR estimate of each study was calculated by the random-effects model. Otherwise, the fixed-effect model was used. The significance of the pooled OR was determined by Z-test. Second, the pooled OR were calculated under dominant model (GG/GA vs. AA), recessive model (GG vs. GA/AA), homozygous model (GG vs. AA), and heterozygous model (GG vs. GA). Last, subgroup analysis was performed by ethnicity for allele comparisons. Sensitivity analyses were carried out to evaluate the influence of each study on the overall estimate from the meta-analysis. Publication bias was assessed using funnel plots, Begg’s test (rank correlation test), and Eggers test (weighted linear regression test for funnel plot symmetry. A χ^2^ test was performed to examine Hardy-Weinberg equilibrium when genotype data were available. *P* < 0.05 was considered to be statistically significant. All analyses were performed by Comprehensive Meta-analysis software version 2.

## Results

### Characteristics of studies

The main characteristics of the 10 studies included in the meta-analysis were summarized in Table 1, of which 8 and 5 studies reported on ESCC and GCA, respectively. A total of 22156 ESCC cases and 28803 controls, 5197 GCA cases and 17613 controls were included. Except for two studies in which GCA and ESCC had the same controls, the other studies measured ESCC and GCA independently. Eight studies were conducted in China, 1 in South Africa, and 1 in US. For the three large GWS studies in Chinese population, samples were collected in different ways. Samples of Abnet et al [11] were collected by Shanxi Upper Gastrointestinal Cancer Genetics Project (Shanxi province) and a prospective cohort of Linxian Nutrition Intervention Trial (Henan province); samples of Wang et al [10] were collected in multiple hospitals throughout the high- and low-incidence areas, and samples of Wu et al [12] were collected in Beijing. The minor allele frequencies in the control groups varied considerably across the ethnicities: 0.217 in Asians, 0.335 in Africa and 0.401 in Caucasian. The genetic distributions of controls in all studies were in Hardy-Weinberg equilibrium.

**Table 1:** Main characteristics of studies included in the meta-analysis.

**Study name(Year)**	**Study type**	**Country**	**Ethics**	**Cancer**	**Cases**	**Controls**
**Abnet(2010)**	GWAS	China	Asian	ESCC	2115	3302
				GCA	1213	3302
**Wang(2010)**	GWAS	China	Asian	ESCC	9053	13283
				GCA	2766	11013
**Wu(2011)[12]**	GWAS	China	Asian	ESCC	8307	8209
**Zhang (2011)**	Case-control	China	Asian	GCA	812	1848
**Bye(2012)**	Case-control	South	Africa	ESCC	672	1707
		Africa				
**Palmer(2012)**	Case-control	United	Caucasian	ESCC	52	210
		States		GCA	122	210
**Hu(2012)**	Case-control	China	Asian	ESCC	1061	1211
**Gu(2012)**	Case-control	China	Asian	ESCC	379	371
**Zhou(2012)**	Case-control	China	Asian	ESCC	517	510
**Wang(2012)**	Case-control	China	Asian	GCA	284	1240

### Esophageal squamous cell carcinoma

**Figure 1:**
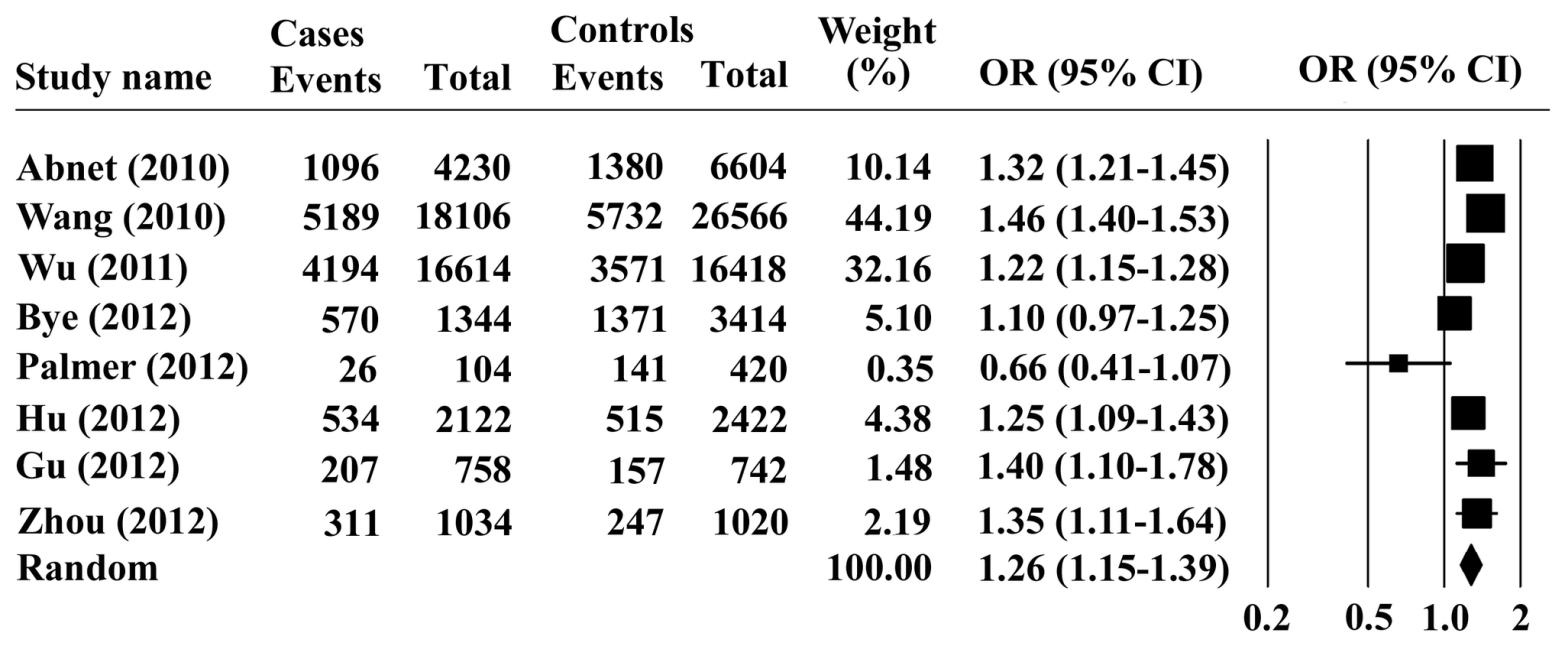
Forest plots for the association of PLCE1 rs2274223 allele G with ESCC.

**Figure 2:**
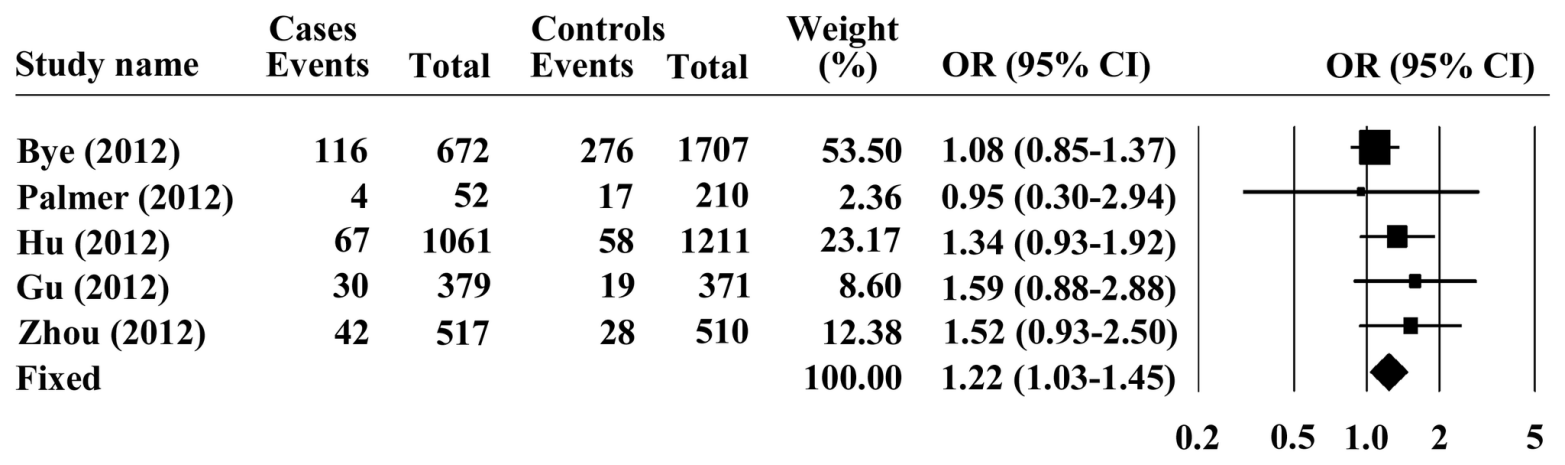
Forest plots for the association of PLCE1 rs2274223 genotype with ESCC under recessive model.

**Figure 3:**
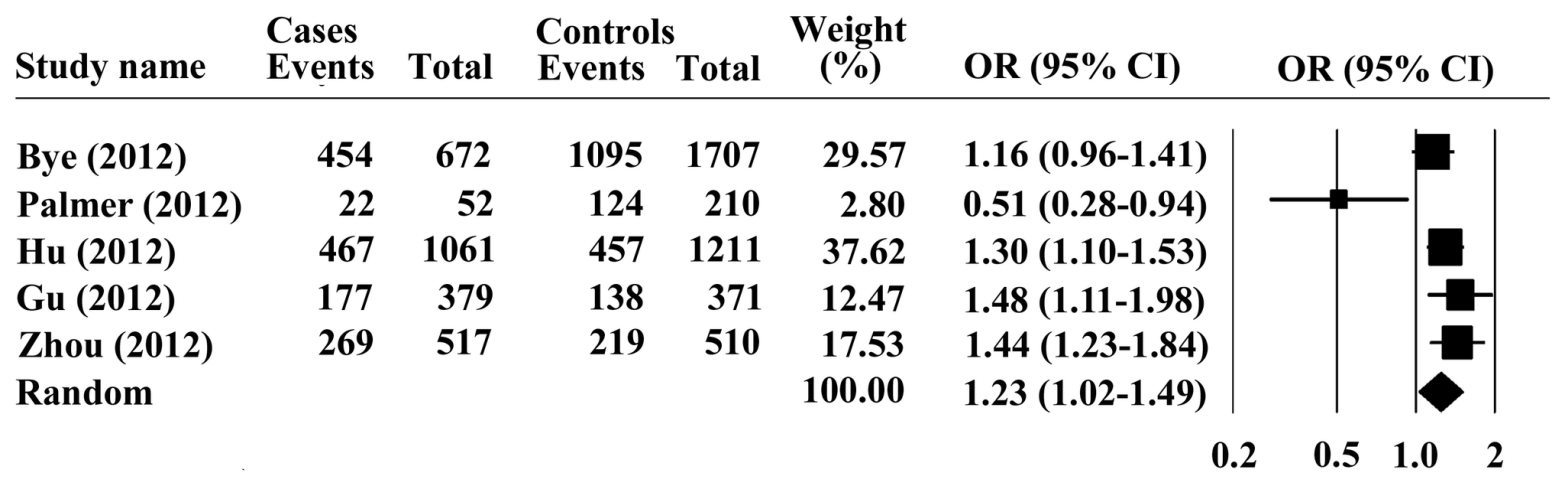
Forest plots for the association of PLCE1 rs2274223 genotype with ESCC under dominant model.

The association of PLCE1 rs2274223 polymorphism and ESCC risk was observed in eight studies. There was a significant heterogeneity in the G versus A allele (Q = 47.45, I^2^=85.25, *P*_*heterogeneity*_ < 0.001) in the studies, therefore, the random model was chosen for representation of the pooled OR value. Overall analysis indicated that the G allele of PLCE1 rs2274223 was significantly associated with increased risk of ESCC, with a pooled OR (95% CI) of 1.26 (1.15-1.39) and *P* < 0.001 for Z test (Figure 1). Furthermore, we investigated the association between the PLCE1 rs2274223 genotype and ESCC risk, assuming dominant and recessive models in five studies. The PLCE1 rs2274223 genotype was associated with ESCC risk, as revealed by the recessive genetic model (GG vs. AG+ AA: OR = 1.22; 95% CI = 1.03–1.45; *P* = 0.024, Figure 2) without genetic heterogeneity (Q = 2.976, I^2^=0, *P*_*heterogeneity*_ = 0.562), dominant model (GG +AG vs. AA: OR = 1.23; 95% CI =1.02-1.49; *P* =0.032 and *P*_*heterogeneity*_ = 0.022, Figure 3), homozygous model (GG vs. AA: OR =1.37; 95% CI =1.11-1.69; *P* =0.003), and heterozygous model (GA vs. AA: OR =1.21; 95% CI =1.01-1.46; *P* =0.049) with substantial heterogeneity. Therefore, G allele or its carriers showed significant increased cancer susceptibility in all genetic models tested.

**Figure 4:**
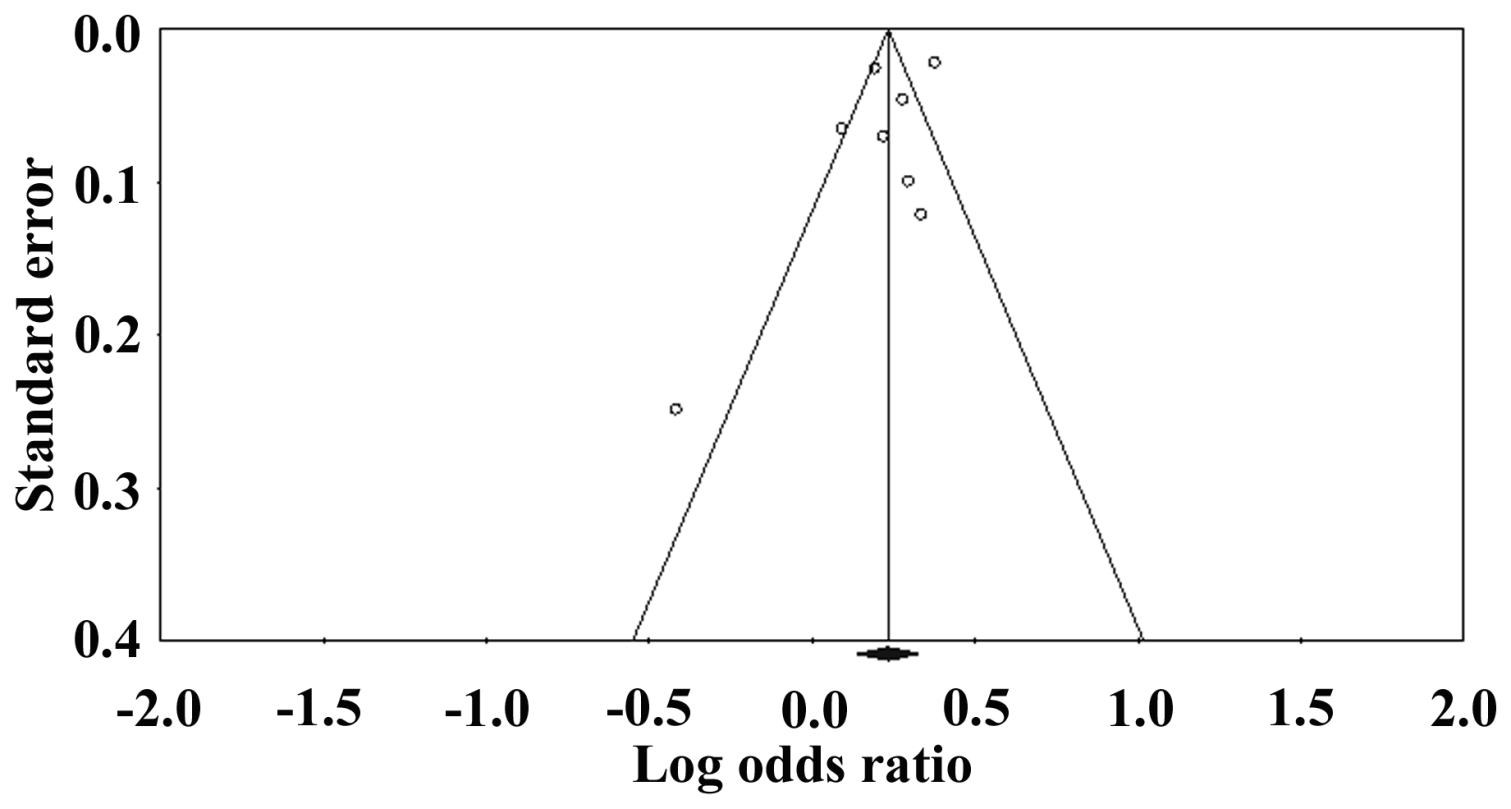
Begg’s funnel plot for estimating the publication bias under PLCE1 rs2274223 allelemodel.

The sensitivity analysis showed no significantly increased or decreased summary OR value while leaving one out at a time, although the findings from Palmer et al were quite influential [18]. The shape of the funnel plot did not reveal any evidence of obvious asymmetry in comparison of the A vs. G allele. Neither Begg’s rank correction (*P* = 0.402) nor Egger’s weighted regression method (*P* = 0.252) showed evidence for publication bias. Begg’s funnel plot for the association is shown in Figure 4.

Subsequently, we stratified studies according to the ethnicity of study subjects. From six studies, ethnic-specific ORs showed that ESCC risk was increased for individuals carrying the G allele compared to those with the A allele in Chinese populations (OR=1.33, 95% CI 1.21–1.45, *P*_*heterogeneity*_ < 0.001) with substantial heterogeneity (Q = 30.41, I^2^=83.56, *P* < 0.001). Similar results were shown by the recessive genetic model (GG vs. AG+ AA: OR =1.44; 95% CI =1.11–1.87; *P* = 0.007 and *P*_*heterogeneity*_ = 0.859), dominant model (GG +AG vs. AA: OR =1.37; 95% CI =1.21-1.55; *P* < 0.001 and *P*_*heterogeneity*_ = 0.659), homozygous model (GG vs. AA: OR =2.56; 95% CI =1.92-3.43; *P* < 0.001), and heterozygous model (GA vs. AA: OR =1.33; 95% CI =1.17-1.52; *P* < 0.001).

### Gastric cardia adenocarcinoma

**Figure 5:**
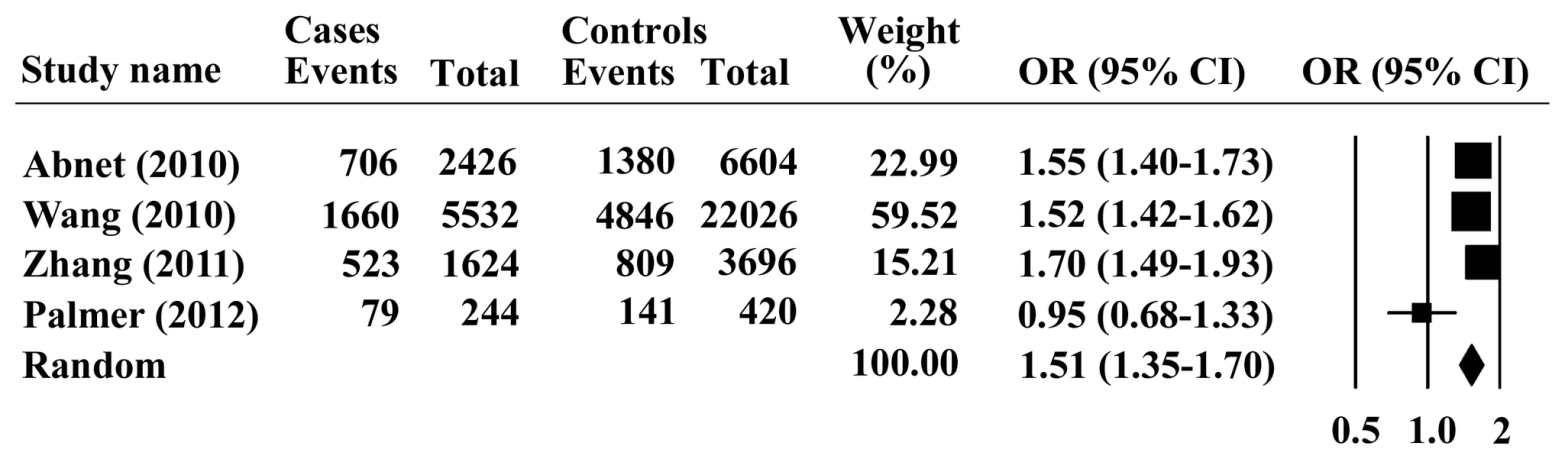
Forest plots for the association of PLCE1 rs2274223 allele G with GCA.

GCA was defined by tumor site in 3 papers. A tumor located in the proximal 3 cm of the stomach was described in Abnet et al study [11]; 2 cm distal to the gastroesophageal junction was used by Wang et al and Zhang et al [10,20]. There was no definition information available from Wang 2012 and Palmer 2012 [18,19]. The heterogeneity was evaluated between each of the studies using Q-test of G versus A allele. Overall significant heterogeneity was detected across five studies (Q = 10.27, I^2^=70.86, *P*_*heterogeneity*_ =0.02), therefore the random model was selected for the pooled odds ratios value. Figure 5 indicated that the summary odds ratios of PLCE1 rs2274223 on the basis of 2968 cases and 9826 controls, a significantly increased GCA risk was observed for G allele in the total population (*P* < 0.001), compared with the A allele with the pooled OR of 1.51 (95% CI: 1.35–1.70).

**Figure 6:**
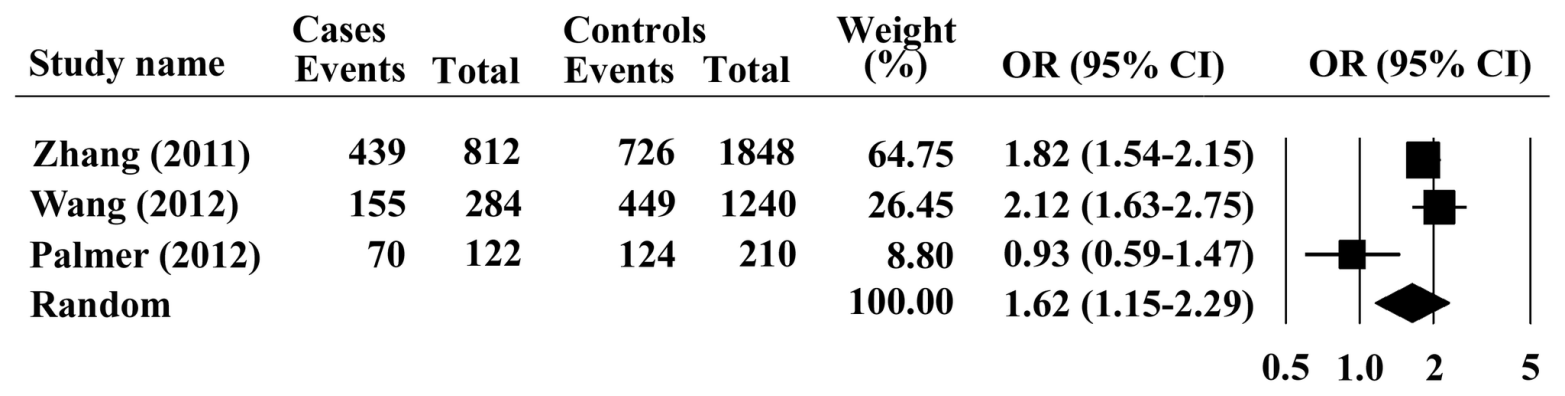
Forest plots for the association of PLCE1 rs2274223 genotype with GCA under dominant model.

The results suggested that no single study significantly affected the pooled ORs in the allele model, indicating that our results were statistically robust. There was no evidence for publication bias using either Begg’s rank correction (*P* = 0.50) or Egger’s weighted regression method (*P* =0.244). The genotype was investigated in three publications, and increased risk of GCA for AG/GG genotypes were observed, comparing with the AA genotype under dominant model (GG +AG vs. AA: OR =1.62; 95% CI =1.15-2.29; *P* =0.006 and *P*_*heterogeneity*_ = 0.008, Figure 6). We found only two studies that provided sufficient genotype data in recessive model, homozygous model and heterozygous models.

When stratified by ethnicity, we focused on the Chinese population from Asian because limited studies were available for meta-analysis from Africa and Caucasian population. A significantly increased GCA risk among subjects carrying G allele in Chinese population was observed (OR =1.56, 95% CI: 1.47-1.64, *P* < 0.001 and *P*_*heterogeneity*_ = 0.340).

## Discussion

In the present study, the results of our meta-analysis indicated that the G allele of PLCE1 rs2274223 variation was significantly associated with increased risk of ESCC and GCA. In addition, subgroup analyses indicated that the G allele significantly increased risk of ECCC and GCA in Chinese population.

GWAS represents an unbiased and fairly comprehensive approach to explore the genetic variation related to cancer. Three independent GWAS recently performed in Chinese populations identified PLCE1 rs2274223 variation as a susceptibility locus for both ESCC and GCA. Replications of the association identified in GWAS by other independent studies were performed, but the results were conflicting. As an important statistical method developed recently, meta-analysis, which can quantitatively combine analyses from different studies, has more statistical power than a single study [23]. To our knowledge, this is the first meta-analysis combining GWAS and replication results to comprehensively evaluate the association between PLCE1 rs2274223 and ESCC and GCA risk.

Overall, we found PLCE1 rs2274223 allele G increased both ESCC and GCA risk with pooled OR (95%CI) of 1.26 (1.15-1.39) and 1.51 (95% CI: 1.35–1.69), respectively. Similar results were obtained from genotype comparison under dominant model (GG +AG vs. AA: OR = 1.23; 95% CI =1.02-1.49 for ESCC; OR =1.62; 95% CI =1.15-2.29 for GCA). Our results further supported that PLCE1 rs2274223 allele G was a common susceptibility locus for ESCC and GCA. Furthermore, the association with PLCE1 rs2274223 allele G was stronger for GCA than for ESCC. Previous efforts characterized common environmental factors between ESCC and GCA, including alcohol consumption, and cigarette smoking [24,25]. In addition, recent evidence has demonstrated that PLCE1 rs2274223 variant was associated with improved gastric cancer patient survival [26]. PLCE1 was over-expressed in ESCC and GCA tumor, compared with in normal tissues (80% versus 36% in ESCC, 72% versus 23% in GCA). PLCE1 contains several Ras binding domains for small G-proteins of the Ras family and is downstream of the Ras superfamily GTPases (Ras, Rap1 and Rap2) involved in regulating cell growth, differentiation, apoptosis and angiogenesis [27–30]. Recently, PLCE1 was considered to play an oncogenic role in intestinal carcinogenesis through inflammation signaling pathways [27]. However, the mechanism of PLCE1 genetic variation on ESCC and GCA susceptibility is unknown; it will therefore be of interest in future studies to investigate the variant and gene functional change and activity, to assess how PLCE1 rs2274223 genotype influences ESCC and GCA risk.

The frequencies of genetic polymorphisms often vary between ethnic groups and are a reflection of genetic diversity in a population. We have noted that the overall frequency of the PLCE1 rs2274223 allele was different in Asians, compared with South Africa and Caucasian in ESCC or GCA patients and controls, suggesting a possible role of ethnic divergence. To test the reliability of our study results, subgroup analyses were performed in terms of ethnicity. We focused on six Chinese studies because the majority of studies were done in Asian population in China. Significant association of PLCE1 rs2274223 variation with ESCC (OR=1.33, 95% CI 1.21–1.45) and GCA (OR =1.56, 95% CI: 1.47-1.64) risk was observed in Chinese population. Under different models, the largest pooled OR was obtained from homozygous model (GG vs. AA: OR =2.56; 95% CI =1.92-3.43; *P* < 0.001). The pooled OR values were higher in Chinese population than in mixed populations under both allele and genotype models, suggesting that the PLCE1 rs2274223 variation has a stronger effect in Chinese population. These findings may in part explain the high rates and the geographic correlation of ESCC and GCA in Chinese population worldwide.

The strengths of this study included the relatively large sample size, no deviation from Hardy–Weinberg equilibrium in controls of all included studies, and low probability of publication bias. But there are some limitations to our primary meta-analysis. First, due to the limited availability of published results, the number of studies included in each meta-analysis was relatively small, and the majority of studies were done in China, while studies among South Africa and Caucasians population were scarce. We expect that as more studies become available, a more accurate estimation of the relationship of PLCE1 rs2274223 variation and ESCC and GCA will be obtained. Second, although we performed the analysis with strict criteria for study inclusion and precise data extraction, significant study heterogeneity existed in all comparisons. Third, our main analysis was based on unadjusted estimates due to the lack of adjusted estimates. Since ESCC and GCA are complex and multifactorial diseases, the exact effect of PLCE1 rs2274223 variation in the context of gene-gene and gene-environment interaction was lacking examination. Therefore, further investigations with larger sample size, strict matching criteria to exclude more confounding factors are warranted to address the possible associations.

In conclusion, this meta-analysis provides evidence supporting an association between PLCE1 rs2274223 variation and genetic risk of ESCC or GCA. However, it is necessary to conduct larger studies in different ethnic populations, with strict selection of patients, and well-matched controls to confirm the association, before PLCE1 rs2274223 variation can be used to prevent ESCC and GCA, and to screen high-risk individuals.

## Supporting Information

Checklist S1 Prisma checklist. (DOC)
